# A Light‐Triggered Mesenchymal Stem Cell Delivery System for Photoacoustic Imaging and Chemo‐Photothermal Therapy of Triple Negative Breast Cancer

**DOI:** 10.1002/advs.201800382

**Published:** 2018-08-24

**Authors:** Chang Xu, Qishuai Feng, Haocheng Yang, Guangxue Wang, Liqun Huang, Qianwen Bai, Chuyi Zhang, Yilong Wang, Yingna Chen, Qian Cheng, Mengwei Chen, Yu Han, Zuoren Yu, Maciej S. Lesniak, Yu Cheng

**Affiliations:** ^1^ The Institute for Translational Nanomedicine Shanghai East Hospital The Institute for Biomedical Engineering & Nano Science Tongji University School of Medicine 150 Jimo Road Shanghai 200120 China; ^2^ Research Center for Translational Medicine Key Laboratory of Arrhythmias of the Ministry of Education of China East Hospital Tongji University School of Medicine 150 Jimo Road Shanghai 200120 China; ^3^ Department of Urology Shanghai East Hospital Tongji University School of Medicine Shanghai 200120 China; ^4^ Institute of Acoustics Tongji University Siping Road 1239 Shanghai 200092 China; ^5^ Department of Neurological Surgery The Feinberg School of Medicine Northwestern University Chicago IL 60611 USA

**Keywords:** light‐controlled release, photothermal therapy, plasmonic–magnetic nanoparticles, stem cells, triple negative breast cancer

## Abstract

Targeted therapy is highly challenging and urgently needed for patients diagnosed with triple negative breast cancer (TNBC). Here, a synergistic treatment platform with plasmonic–magnetic hybrid nanoparticle (lipids, doxorubicin (DOX), gold nanorods, iron oxide nanocluster (LDGI))–loaded mesenchymal stem cells (MSCs) for photoacoustic imaging, targeted photothermal therapy, and chemotherapy for TNBC is developed. LDGI can be efficiently taken up into the stem cells with good biocompatibility to maintain the cellular functions. In addition, CXCR4 on the MSCs is upregulated by iron oxide nanoparticles in the LDGI. Importantly, the drug release and photothermal therapy can be simultaneously achieved upon light irradiation. The released drug can enter the cell nucleus and promote cell apoptosis. Interestingly, light irradiation can control the secretion of cellular microvehicles carrying LDGI for targeted treatment. A remarkable in vitro anticancer effect is observed in MDA‐MB‐231 with near‐infrared laser irradiation. In vivo studies show that the MSCs‐LDGI has the enhanced migration and penetration abilities in the tumor area via both intratumoral and intravenous injection approaches compared with LDGI. Subsequently, MSCs‐LDGI shows the best antitumor efficacy via chemo‐photothermal therapy compared to other treatment groups in the TNBC model of nude mice. Thus, MSCs‐LDGI multifunctional system represents greatly synergistic potential for cancer treatment.

## 1 Introduction

Breast cancer is a life‐threatening disease with the highest incidence and mortality in women with cancers.^1^ In 2012, there were more than 1.7 million newly diagnosed cases and 521 900 deaths worldwide.^2^ Triple negative breast cancer (TNBC), accounting for 15–20% of breast cancer, is characterized by lacking the overexpression of estrogen receptors, progesterone receptors, and human epidermal growth factor receptor 2 (HER2).^3^, ^4^ With the highest rate of metastasis and frequent recurrence,^5^ TNBC is the most difficult subgroup to target and cure compared to other forms of breast cancer. Besides surgical resection, chemotherapy agents such as doxorubicin (DOX) and paclitaxel are often recommended for breast cancer treatment. However, severe systemic toxicity, drug resistance, and poor selectivity of therapeutic agents to the breast cancer tissues especially the metastasis sites are the major issues limiting the chemotherapy efficacy.^6^ Although liposomal formulations such as DOXIL have been developed to improve the therapeutic efficacy to the tumors,^7^ active accumulation and controlled drug release to the cancer tissues remain unsolved. Lacking the specific targeted therapy is the bottleneck for TNBC treatment.

Light‐based therapeutic approaches with unique features such as spatiotemporal control, minimal invasiveness, and high selectivity have received tremendous attention for targeted cancer treatment. Photothermal therapy (PTT),^8^ where light is converted to heat via photothermal agents for thermal ablation, is a noninvasive therapy for breast cancer treatment. Versatile platforms have emerged in this field to generate heat under light irradiation, including organic compounds such as indocyanine green, graphene oxide nanoparticles, carbon nanotubes, as well as metallic nanomaterials with anisotropic structures.^9^ Among all these nanoparticle platforms, gold nanoparticles with unique optical properties and good biocompatibility are widely investigated as the PTT agents.^10^ For instance, the surface plasmon resonance absorption and light conversion efficiency of gold nanoparticles such as gold nanorods (GNRs) and nanoshells can be tuned to the near‐infrared (NIR) region by adjusting their anisotropic structures.^11^, ^12^ PTT in combination with chemotherapy and radiotherapy have shown the capability to inhibit the primary tumor growth and cell invasion in cancer with a relatively low drug administration dose.^13^, ^14^ The efficacy is highly dependent on the transportation of PTT agents to the cancerous tissues.

Stem cells with the intrinsic tumor tropism have attracted huge interests as the emerging biovehicles to deliver therapeutic agents for cancer treatment.^15^, ^16^, ^17^, ^18^, ^19^, ^20^ Studies show that chemokines such as stromal cell–derived factor‐1 (SDF‐1) are important factors to facilitate stem cell migration to lesion locations via the specific receptors like CXCR4.^21^, ^22^ Hypoxic‐treated neural stem cells showed the overexpression of CXCR4 and could enhance the tumor migration ability.^23^ There were numerous studies utilized the homing ability of stem cells for tumor targeting.^24^, ^25^, ^26^, ^27^, ^28^ In addition, it has been reported that iron oxide nanoparticles with the released iron ions could increase the CXCR4 expression on the mesenchymal stem cell (MSC) surface and promote the migration ability.^29^ Direct loading anticancer drugs may have severe toxic effects on stem cells which could hamper the cell viability and migration ability to cancer cells. Although biomolecules such as antibodies and proteins can be engineered to load with stem cells, forming teratoma is the major hurdle remaining with their differentiation capability after transplantation.^30^

**Scheme 1:**
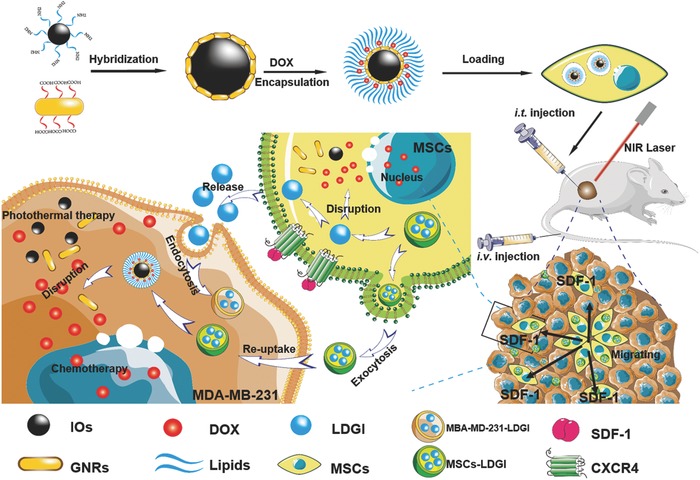
The scheme of LDGI‐loaded MSCs for enhanced tumor migration ability, PTT, and chemotherapy of TNBC.

Stem cells combined with stimuli‐responsive therapeutic agents are promising with the hope that they could achieve controlled destruction and prevent teratoma formation for cancer treatment. In this work, we design a novel therapeutic platform based on the MSC loaded with the plasmonic–magnetic nanoparticles composed of lipids, DOX, gold nanorods, and iron oxide nanoclusters (LDGI) to achieve the tumor‐tropic migration and multifunctional modalities of photoacoustic imaging, PTT, and chemotherapy for TNBC (**Scheme** **1**). Gold nanorods are selected as the PTT agent due to their high photothermal conversion efficiency and photoacoustic imaging for cell tracking.^31^, ^32^, ^33^ The platform is further combined with iron oxide nanoclusters, which could provide a scaffold to assemble gold nanorods and offer the iron source for enhancing CXCR4 expression of MSCs. Additionally, DOX is encapsulated into the lipid system on the nanoparticles by physical adsorption as the chemotherapeutic agent. The MSCs vehicles could not only enhance the distribution of LDGI in tumor by intratumoral injection, but also accumulate in the tumor area more efficiently than LDGI alone via intravenously injection. Moreover, MSCs‐LDGI could release microvesicles containing LDGI along migration process that could be further taken up by cancer cells. The LDGI‐carried MSCs were NIR light sensitive and could selectively release DOX and GNR to damage the TNBC cells via the synergistic effect of PTT and chemotherapy. To the best of our knowledge, this is the first example of utilizing the plasmonic–magnetic nanostructures to externally control the therapeutic agent release in stem cells for combination functions of photoacoustic imaging, PTT, and chemotherapy.

## 2 Results and Discussion

### 2.1 Synthesis and Characterization of LDGI

**Figure 1:**
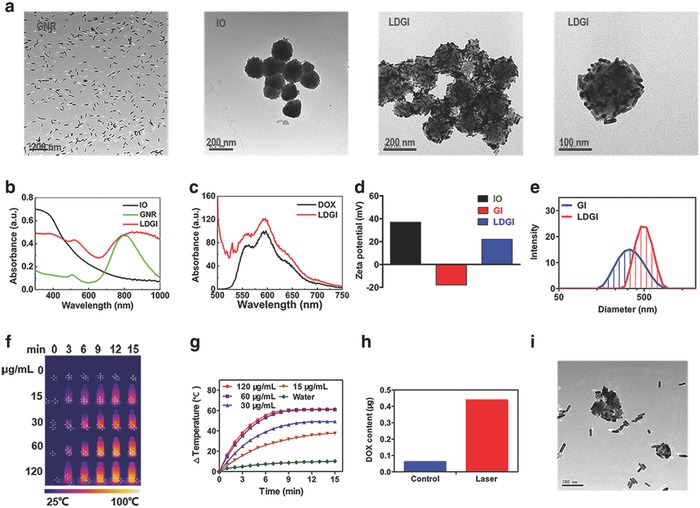
Characterization of LDGI. a) Representative TEM images of GNR, IO, and LDGI. b) UV–vis spectra of IO, GNR, and LDGI. c) Fluorescence spectra of LDGI and DOX (1 µg mL^−1^). d) Zeta potential of IO, GNR, and LDGI in deionized water. e) Size distribution of GI and LDGI in deionized water. f) Photothermal effect of LDGI at different concentrations and g) the corresponding temperature changing curves under 808 nm laser at 3 W cm^−2^ for 15 min. h) Drug release behavior of the LDGI aqueous solution with laser irradiation (3 W cm^−2^, 3 min). i) The TEM image of LDGI post irradiation.

The GNRs with the aspect ratio of 5 were prepared via the one‐step seedless method.^34^ Bifunctional HS—PEG)—COOH was utilized to modify GNR to achieve stability and functionality. GNRs were conjugated on the polyethyleneimine‐coated iron oxide (IO) nanoparticles via the amide bond to form the plasmonic–magnetic nanostructure. LDGI were synthesized via the reverse phase evaporation method to incorporate GNR–IO (GI), DOX, 1,2‐dioleoyl‐3‐trimethylammoniumpropane (DOTAP), 1,2‐distearoyl‐*sn*‐glycero‐3‐phosphocholine (DSPC), and 1,2‐distearoyl‐*sn*‐glycero‐3‐phosphoethanolamine‐*n*‐[(carboxy(polyethyleneglycol)_2000_] (ammonium salt) (mPEG_2000_‐DSPE).^35^, ^36^, ^37^ The shape and size of the prepared nanoparticles were determined by transmission electron microscopy (TEM). The synthesized GNR had a mean length of 40 ± 5 nm and width of 8 ± 3 nm (**Figure** **1**a). The IO had an average diameter of 140 ± 10 nm. Comparing with IO, the size distribution of LDGI shifted slightly to 160 ± 10 nm. Both of IO and GNR could be clearly observed in the hybrid nanostructures (Figure 1a). GNR functionalized with —COOH were assembled on the IO surface with positive charges due to the formation of the amide bond and electrostatic interaction. It could be observed that GNR had a transverse plasmonic absorption at 510 nm and a longitudinal plasmonic band at 800 nm, which were consistent to the rod‐like nanostructures (Figure 1b). The LDGI had the characteristic absorptions of GNR and IO. Interestingly, a broad plasmonic absorption at 830 nm with a 30 nm redshift compared with GNR was observed, which might be due to plasmonic coupling of GNR packed on the IO.^38^, ^39^ It indicated that the hybrid nanostructure of IO and GNR was successfully achieved and presented the physical properties of both GNR and IO. The Au and Fe in LDGI were further quantified by inductively coupled plasma mass spectrometry (ICP‐MS), showing that the ratio of the number of atoms of gold to iron in LDGI was 1.08 (Figure S1, Supporting Information). In addition, the emission peak at 590 nm representing the DOX fluorescence was observed in the LDGI aqueous solution, suggesting that the DOX was encapsulated into the LDGI (Figure 1c). The DOX loading efficiency of LDGI was 1.3 ± 0.3% w/w, and the encapsulation efficacy of DOX in LDGI was 18.7 ± 2.1%. Since the cationic lipid DOTAP was introduced to enhance the interaction with the cellular carriers, the surface charge property of LDGI was analyzed (Figure 1d). The zeta potential of polyethyleneimine (PEI)‐coated IO was +37 mV and the GI modified with HS—PEG—COOH was −19 mV. The LDGI had a positive zeta potential at +20 mV, confirming the successful cationic lipid coating (Figure 1d). LDGI could be well dispersed in the aqueous solution, as proved by dynamic light scattering (DLS) analysis (Figure 1e).

The photothermal ability of LDGI was investigated under the laser irradiation at 808 nm at different concentrations (Figure 1f,g). Water had minimal absorption at 808 nm and showed little temperature increase during 15 min irradiation. The temperature of LDGI aqueous solution was elevated 37.6 °C above the original temperature at 15 µg mL^−1^. When the LDGI concentration further increased to 120 µg mL^−1^, the photothermal effect of LDGI was more pronounced to heat the aqueous solution. It was also found that the photothermal effect of LDGI could be tuned by the laser power. With increasing the laser intensities, the heat generation of LDGI was enhanced, as reflected by the temperature changing curves (Figure S2a,b, Supporting Information). And, the synthesized LDGI possessed stable heat conversion ability after 4 times irradiation (Figure S3, Supporting Information). The cumulative DOX release was evaluated by fluorescence spectra (Figure S4, Supporting Information). The LDGI had a burst release in the first 0.5 h and after that the drug was slowly released. After 14 h, the free DOX released into the solution from LDGI reached a platform and the release amount was 23% of the total. In addition, the NIR light–triggered drug release was carried out in phosphate buffer solution (PBS) at room temperature. After 808 nm laser irradiation for 3 min, the temperature of LDGI solution increased to 61 °C and the released drug was 0.4 µg mL^−1^ that was 6.8‐fold higher compared with the control group (Figure 1h). It indicated that the hybrid structure of LDGI could be responsive to heat and rapidly dissociated to release DOX. Indeed, LDGI was disassembled and free GNRs were observed post irradiation, as confirmed via TEM (Figure 1i). It suggested that the light‐triggered disassembly and DOX release could be utilized for controlled cell destruction. Furthermore, the stability and toxicity of DOX after laser irradiation were also examined.^40^ Different power densities (1.5, 3, and 4 W cm^−2^) and exposure time periods (0, 5, 10, 15 min) were utilized to investigate the laser irradiation impact (Figure S5, Supporting Information). It could be observed that the absorption and fluorescence spectra of DOX after irradiation were similar to the free DOX. And, the cytotoxicity of laser‐irradiated DOX was also close to the free DOX (Figure S6, Supporting Information). It could be concluded that laser irradiation did not impact the stability and cytotoxicity of DOX to MDA‐MB‐231.

### 2.2 LDGI‐Loaded MSCs Maintained the Tropism to Cancer Cells

**Figure 2:**
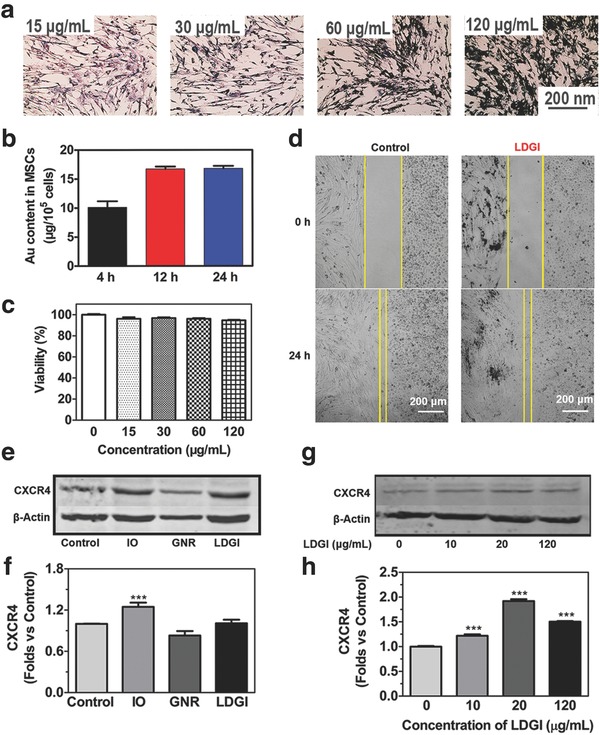
Loading LDGI into MSCs and the migration ability of the combination platform. a) Prussian blue stain of MSCs treated with different concentrations of LDGI (15, 30, 60, 120 µg mL^−1^) after 24 h incubation. b) ICP‐MS quantification of LDGI at the incubation times of 4, 12, and 24 h. c) Cell viability with different concentrations of LDGI. d) MSCs migration toward MDA‐MB‐231 cells treated with 25 µg mL^−1^ LDGI and without LDGI in 24 h. e) Western blot analysis of CXCR4 expression in MSCs after treatment with nanoparticles including IO, GNR, and LDGI. f) Quantification of the western blotting results in (e). g) Specific surface markers of stem cells expression on MSCs treated with different concentrations of LDGI and h) the quantification of western blotting results by measuring the band density and then normalizing it to β‐actin.

Loading of LDGI into MSCs without changing the migratory ability is the key for stem cell–targeted delivery. In order to investigate the cell loading capability, LDGI with different concentrations were incubated with MSCs for 24 h. All the MSCs could internalize LDGI and the nanoparticle loading capacity could be enhanced by increasing the LDGI concentration, as shown via Prussian blue staining (**Figure** **2**a). The amount of LGDI in MSCs was quantified at different incubation times by ICP‐MS. LDGI was gradually accumulated into MSCs over time and reached the maximum loading of 16.83 µg Au per 10^5^ MSCs at 24 h (Figure 2b). The biocompatibility of LDGI in MSCs was investigated by the cell counting kit‐8 (CCK‐8) assay (Figure 2c). No significant toxicity from LDGI was observed and over 93% of MSCs survived after loading (Figure 2c). The influence of LDGI on MSCs was further evaluated through flow cytometry. The cells were characterized using six representative surface markers consisting of four positive markers including CD29, CD44, CD90, and CD105 and two negative markers of CD34 and CD45. Compared with MSCs, the LDGI‐loaded MSCs showed similar expression of the positive surface markers and nonexpression of CD 34 and CD 45, indicating that LDGI did not influence the normal function of MSCs (Figure S7, Supporting Information).

A critical feature of MSCs used as biovehicles is their tropic migration to cancer cells. The migration property of the LDGI‐loaded MSCs was examined via the wound healing assay. Similar to MSCs, LDGI‐loaded MSCs could migrate toward to the MDA‐MB‐231 cells and the gap between the stem cells and cancer cells was merged after 24 h (Figure 2d). Furthermore, CXCR4, a chemokine receptor, could specifically recognize the SDF‐1 overexpressing in the tumor tissue. The protein expression of CXCR4 was examined through western blot (Figure 2e). Compared with the control group, IO and LDGI group played positive roles in CXCR4 expression, with 1.3‐fold and 1.2‐fold increase of CXCR4 expression, respectively (Figure 2f). In contrast, the GNR group did not promote CXCR4 expression. The protein expression level of CXCR4 was also elevated with increasing of LDGI concentrations (Figure 2g). At 20 µg mL^−1^ of LDGI incubation condition, the CXCR4 level was 1.9 times higher compared with the control group (Figure 2h). It was reported that iron‐based nanoparticles could significantly enhance the expression of CXCR4 and promote MSC homing.^24^ Consistently, our results showed that the LDGI had the potential of facilitating the migration ability of MSCs via upregulation of CXCR4 induced by iron‐containing nanoparticles.

### 2.3 Photothermal‐Triggered Drug Release in LDGI‐Loaded MSCs

**Figure 3:**
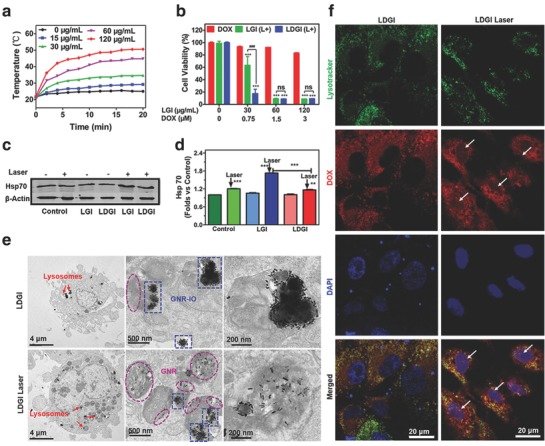
NIR light–triggered DOX release and photothermal effect of LDGI. a) Temperature rising curve of MSCs incubated with LDGI at different concentrations (0, 30, 60, 120 µg mL^−1^) with laser irradiation (3 W cm^−2^, 20 min). b) Cell viability of MSCs incubated with DOX, LGI, and LDGI nanoparticles for 24 h and treated with NIR laser (3 W cm^−2^, 5 min). c) Western blot analysis of Hsp70 protein expression in MSCs after treatment with nanoparticles including LGI and LDGI post laser irradiation (1.5 W cm^−2^, 5 min) and d) the quantification of western blotting results by measuring the band density and then normalizing according to β‐actin. e) Representative TEM images of LDGI‐loaded MSCs treated with and without laser (3 W cm^−2^, 5 min). f) Confocal images of DOX release from LDGI in MSCs before and after laser (3 W cm^−2^, 5 min). Green fluorescence represents the lysotracker green accumulated in lysosomes, red fluorescence represents DOX trapped in LDGI and free DOX, and blue fluorescence represents 4′,6‐diamidino‐2‐phenylindole (DAPI) in the cell nucleus.

Combination treatment approaches were proved to be more efficient over the single treatment modality for cancer treatment.^41^, ^42^, ^43^ The heat generation via LDGI‐loaded MSCs under irradiation was LDGI concentration dependent with a significant increase in temperature at the concentration of 120 µg mL^−1^ LDGI (**Figure** **3**a). The cell culture medium temperature could increase from room temperature to 43 °C post 5 min under NIR exposure and could be further elevated with prolonged irradiation time. MSCs alone had little NIR light absorption and the temperature only increased 3.6 °C in the MSC control group. The results demonstrated that LDGI‐loaded MSCs maintained the photothermal property. Then, the cytotoxicities of DOX, lipid@GNR–IO (LGI), and LDGI post irradiation were investigated with the consideration of minimizing the potential risk of tumorigenicity of MSCs. After NIR exposure, the cell viability post NIR irradiation was quantified via the CCK‐8 assay. MSCs could be efficiently damaged after laser treatment (Figure 3b). No toxicity effect was observed in the cell control group with irradiation only. Both of LGI and LDGI showed good cytotoxicity and over 90% of cells were destructed at 60 and 120 µg mL^−1^ of nanoparticles. It should be noted that at the corresponding LDGI concentrations, DOX only showed moderate cytotoxicity with only 7.5% at 60 µg mL^−1^ and 18% at 120 µg mL^−1^, respectively. Synergistic effect of PTT and chemotherapy via LDGI was observed at 30 µg mL^−1^ of LDGI. Over 80.3 ± 2% of cells could be damaged via LDGI with NIR irradiation, while LGI induced 31.9 ± 4.2% cytotoxicity and 6.5 ± 0.74% for DOX. The 70 kDa heat shock proteins (Hsp70) was a product of cells protection mechanism from heat or stress.^44^, ^45^ Next, the Hsp70 expression was examined to evaluate the synergistic effect using western blot (Figure 3c). The Hsp70 expression of MSCs, MSCs‐LGI, and MSCs‐LDGI groups would be upregulated to 1.20, 1.73, and 1.16 times after laser irradiation contrast with the control groups (Figure 3d). The introduction of DOX reduced the expression of Hsp70 protein, which could contribute to the higher cytotoxicity of LDGI.

As mentioned above, the LDGI nanoparticles were designed to be photothermal responsive. The laser‐responsive controlled release of LDGI was further investigated by TEM. Most LDGI maintained the hybrid structure and localized in cell lysosomes without irradiation. Consistently, no LDGI was detected in the cell nuclei. The heat generated by LGDIs upon irradiation initiated the disassembly of LDGI, being shown by the free GNR scattered in the cell lysosomes (Figure 3e). With laser irradiation, the heat generated by GNR would destroy the hybrid nanostructure and trigger the DOX release. As shown via confocal microscopy, majority of LDGI were localized in lysosomes of MSCs, as indicated via the DOX fluorescence (Figure 3f). Little drug fluorescence was observed in the cell nucleus, suggesting that the LDGI had good stability to encapsulate DOX in the hybrid nanostructures. Post 2 h of NIR irradiation, DOX fluorescence signal was observed in the cell nucleus, confirming the photothermal responsibility of LDGI for drug release. The results indicated that the light‐responsive LDGI could not only induce the cell death via hyperthermia effect but also promote the controlled drug release to achieve a synergistic efficacy combined with PTT and chemotherapy, which could further reduce the drug dose and side effects of DOX.

### 2.4 In Vitro Anticancer Effect of MSCs‐LDGI

**Figure 4:**
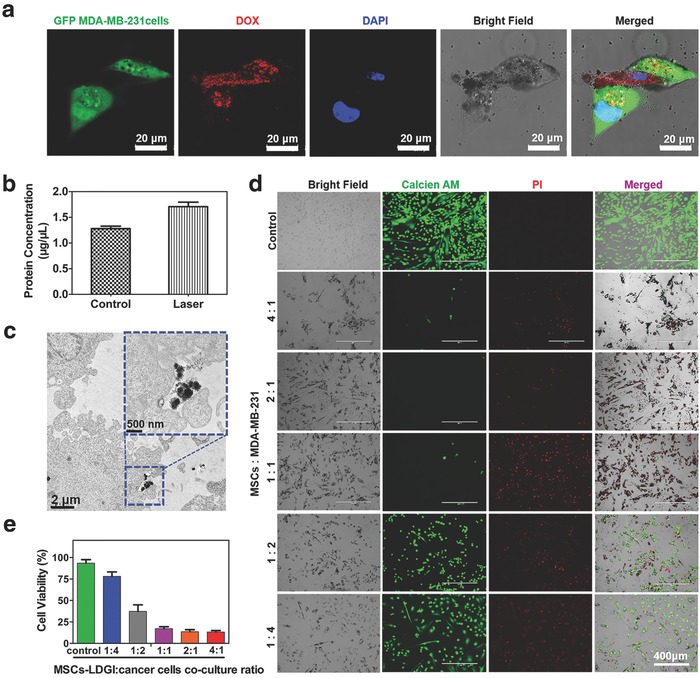
The anticancer effect of MSCs‐LDGI against MDA‐MB‐231 cells. a) Confocal images of green florescence protein (GFP)‐labeled MDA‐MB‐231 cells cocultured with MSCs‐LDGI (green florescence represents MDA‐MB‐231 cells, red florescence represents DOX trapped in LDGI, and blue florescence represents the cell nucleus). b) Protein quantification of cells culture medium after laser irradiation (1.5 W cm^−2^, 5 min). c) TEM image of exocytosis of LDGI from MSCs‐LDGI post 24 h incubation. d) LIVE/DEAD cell viability tests of MSCs‐LDGI cocultured with MDA‐MB‐231 cells post laser irradiation (green fluorescence represents live cells stained with calcein‐AM solution and red fluorescence represents dead cells stained with propidium iodide (PI) solution). e) Cell viability of MSCs‐LDGI and MDA‐MB‐231 cells with different ratios for 24 h with and without irradiation tested by CCK‐8 kit after 24 h.

To evaluate the antitumor efficacy of MSCs‐LDGI, MSCs‐LDGI were cocultured with MDA‐MB‐231 cells for PTT in vitro. To visualize the transportation of LDGI from the stem cells to the cancer cells, the MSCs‐LDGI were cocultured with green‐fluorescence MDA‐MB‐231 cells and examined by confocal imaging after 24 h. As a result, nanoparticles with red fluorescence were observed in both MSCs and green fluorescence MDA‐MB‐231 cells (**Figure** **4**a). This phenomenon revealed that MSCs served as a LDGI vehicle to deliver the nanoparticle into tumor cells. Furthermore, the intracellular behavior of LDGI could be regulated by the laser irradiation. The amount of microvesicles secreted by MSCs‐LDGI could be enhanced 1.6 times after laser irradiation, contrasting with the control group (Figure 4b). This could be further illustrated by TEM. LDGI was excreted into the supernatant from MSCs post laser irradiation (Figure 4c). The excreted protein of extracellular vesicles was analyzed via sodium dodecyl sulphate‐polyacrylamide gel electrophoresis (SDS‐PAGE) assay. The stripes of microvesicles (MVs) from MSCs‐LDGI were consistent with the MVs from MSCs (Figure S8, Supporting Information). In addition, the western blot analysis was used to examine the specific proteins expressed by LDGI MVs. The result showed that the exosome proteins Alix and CD63 were found on the LDGI MVs (Figure S9, Supporting Information). And, the characterization was conducted on the microvesicles from MSCs‐LDGI (Figure S10, Supporting Information). Then, the cell viabilities of cocultured MSCs‐LDGI and MDA‐MB‐231 cells with laser were studied. After laser irradiation (3 W cm^−2^) for 5 min, LIVE/DEAD staining and CCK‐8 test were performed after incubated for another 24 h. The LIVE/DEAD staining result showed the anticancer efficacy of MSCs‐LDGI vividly. The cell viability of different groups had shown a MSCs‐LDGI amount–dependent decrease. When the ratios of MSCs‐LDGI to MDA‐MB‐231 cells were 4:1, 2:1, and 1:1, almost all the cells were destroyed and stained by propidium iodide (PI). As for the ratio of 1:2 and 1:4, there were a large number of live cells stained by calcein (Figure 4d). And, the cytotoxicity of cocultured cells was further quantified by CCK‐8 assay. The cell toxicity of MSCs‐LDGI:MDA‐MB‐231 cells of 4:1, 2:1, 1:1, 1:2, 1:4 were 13.4 ± 1.2%, 13.7 ± 2%, 17.2 ± 2.3%, 37.4 ± 8.7%, and 78.2 ± 11.6%, respectively. With the increased MSCs‐LDGI to MDA‐MB‐231 cell ratios, efficacious antitumor effect could be achieved with the ratios of MSCs‐LDGI:MDA‐MB‐231 cells of 4:1, 2:1, and 1:1 (Figure 4e). Furthermore, the LDGI MVs were collected and incubated with MDA‐MB‐231. The DOX uptake in the cancer cells was tested by flow cytometry. The cancer cells showed fluorescence of DOX after incubated with different concentrations of LDGI MVs. The DOX positive cells were increased to 12.6% after incubated with 20 µg mL^−1^ MVs (Figure S11, Supporting Information). The cytotoxicity of LDGI MVs was further studied. The cancer cells could be eradicated after incubated with LDGI MVs with laser irradiation, as stained by PI (Figure S12, Supporting Information). Our results showed that not only the MSC encapsulated with LDGI could effectively destruct the cancer cells via the chemo‐photothermal therapy, the LDGI MVs could also act as therapeutic agents for chemo‐photothermal therapy.

### 2.5 Intratumoral Distribution and In Vivo Antitumor Effect of MSCs‐LDGI with Intratumoral Injection

**Figure 5:**
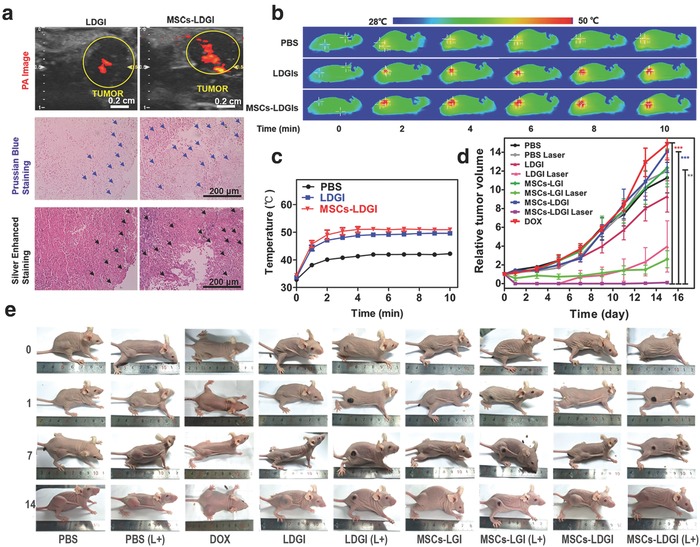
In vivo antitumor effect of MSCs‐LDGI (intratumoral injection). a) In vivo photoacoustic image, Prussian blue staining, and silver‐enhanced staining of tumor tissues of LDGI and MSCs‐LDGI, respectively. b) Infrared thermal images and c) temperature increasing curves of PBS, LDGI, MSCs‐LGI, and MSCs‐LDGI with continuous laser irradiation of 808 nm laser (1.5 W cm^−2^, 10 min) in vivo. d) Relative tumor volume and e) images of mice of being intratumorally injected with DOX, PBS, LDGI, MSCs‐LGI, MSCs‐LDGI with and without NIR laser irradiation within 15 days.

The studies had confirmed the synergistic therapeutic efficacy of MSCs‐LDGI in vitro, it is encouraging to investigate the in vivo antitumor effect. The intratumor distribution of MSCs‐LDGI was assessed by photoacoustic (PA) imaging. In the PA image, the distribution area of MSCs‐LDGI was 4.2 times larger than LDGI nanoparticles alone, illustrating that MSCs‐LDGI maintained the ability of MSCs to migrate through the tumor. Consistent with PA imaging results, the histology studies further confirmed the broader distribution area of MSCs‐LDGI. The IOs in LDGI were stained by Prussian blue kit and GNRs were stained by silver enhancement staining, respectively. All the staining results showed the MSCs‐LDGI had a broader distribution area than LDGI alone, attributed to the migration ability of MSCs (**Figure** **5**a). Encouraged by the in vitro PTT effect and tumor‐targeted distribution, the therapeutic effect of MSCs‐LDGI was evaluated in MDA‐MB‐231 tumor bearing mice. The mice were treated with laser irradiation 3 days after intratumoral injection (1.5 W cm^−2^, 10 min). The tumor surface temperature was monitored and recorded (Figure 5b). The temperature increasing curves showed that LDGI, MSCs‐LGI, and MSCs‐LDGI groups could reach about 51.9 ± 0.8 °C, while the temperature of PBS group was only 42 ± 0.6 °C after 10 min irradiation (Figure 5c). To assess the antitumor efficacy, the tumor size and mice body weight were continuously measured in the next 15 days. The body weights of mice showed no significant difference in all the experimental groups (Figure S13, Supporting Information). The tumor volume of mice treated with PBS, DOX, and LDGI, MSCs‐LGI, MSCs‐LDGI without laser irradiation kept increasing after being administrated. The dose of DOX used in our designed system was 0.06 mg kg^−1^ for the intratumoral injection, which was equivalent to the amount of DOX in the MSCs‐LDGI group to achieve the synergistic treatment effect of MSCs‐LDGI. The dose of DOX was much lower than the commonly used local injection doses (range 0.25–7.5 mg kg^−1^) in cancer treatment due to the combination of chemo‐photothermal therapy.^46^, ^47^, ^48^, ^49^ Therefore, the effect of DOX alone at this low dose of DOX was negligible. The groups of LDGI, MSCs‐LGI, and MSCs‐LDGI with laser irradiation showed significant anticancer effects. Among the treated groups, MSCs‐LDGI was the most efficient approach to inhibit the tumor growth and recurrence which could be attributed to the better distribution and synergistic effect (Figure 5d). The group of MSCs‐LDGI with laser irradiation exhibited the greatest antitumor efficiency among all the groups for TNBC treatment. After 14 days post laser irradiation, the picture for each mouse was recorded before being executed. In the mice images, tumor recurrence could be observed in the laser‐treated groups of LDGI and MSCs‐LGI. The laser irradiation could partially inhibit the tumor growth compared to the group without laser irradiation (Figure 5e). Only the MSCs‐LDGI with laser group showed the complete tumor eradication and no recurrence was observed during the 2 week experimental period. It indicates that the intratumoral distribution of therapeutic agents is a key factor to achieve good antitumor efficacy. Our results revealed that MSC‐mediated delivery was an effective strategy to achieve broad distribution for therapeutic agents including nanomedicines at the tumor site. Thus, the designed LDGI‐loaded MSCs was proved to be an effective TNBC treatment agent.

### 2.6 Biodistribution and In Vivo Antitumor Effect of MSCs‐LDGI with Intravenous Injection

**Figure 6:**
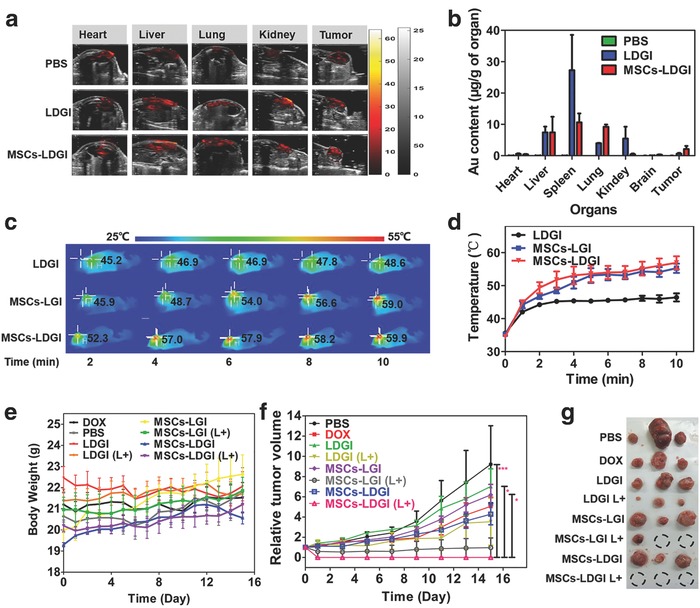
In vivo antitumor effect of MSCs‐LDGI via intravenous injection. a) In vivo photoacoustic image of mice organs of MSCs‐LDGI, LDGI, and PBS, respectively. b) Biodistribution of gold amount in heart, liver, spleen, lung, kidney, brain, and tumor tissues post 3 days of intravenous injection. c) In vivo infrared thermal images and d) the corresponding temperature increasing curves of LDGI, MSCs‐LGI, and MSCs‐LDGI with continuous laser irradiation of 808 nm laser (1.5 W cm^−2^, 10 min). e) Body weight curves of mice injected with DOX, PBS, LDGI, MSCs‐LGI, MSCs‐LDGI with and without NIR laser. f) Relative tumor volumes and g) the image of tumors from the mice treated with DOX, PBS, LDGI, MSCs‐LGI, MSCs‐LDGI with and without NIR laser irradiation post 15 days.

The tumor tropical efficiency and therapeutic effect of MSC–LDGI via intravenous administration was further investigated. First, the in vivo biodistribution study of MSCs‐LDGI was carried out by PA imaging post intravenous injection (**Figure** **6**a). As shown in the PA images, both of LDGI and MSCs‐LDGI could accumulate in liver, lung, kidney, and tumor tissues. Little PA signals were observed in the heart. Both the groups showed strong signals in the liver. Compared with LDGI‐treated group, the MSCs‐LDGI‐treated mouse showed higher PA signals in the lung and tumor area and less accumulation in the kidney. According to the PA images, MSCs‐LDGI could more likely accumulate in the tumor tissues compared with LDGI by enhanced permeability and retention (EPR) effect.

The content of nanoparticles in the organs was quantified via ICP‐MS (Figure 6b). The mouse tissues including heart, liver, spleen, lung, kidney, brain, and tumor were collected post 3 days of intravenous injection of PBS, LDGI, and MSCs‐LDGI, respectively. The PBS group was chosen as the control for quantification of gold content. For the LDGI‐treated mice, spleen tissue showed the highest accumulation of gold (27.30 µg g^−1^ of tissue). Liver (7.41 µg g^−1^ of tissue), kidney (5.46 µg g^−1^ of tissue), and lung (3.94 µg g^−1^ of tissue) tissues also had high content of gold. 0.63 µg g^−1^ of tissue of gold was accumulated in the tumor tissue. Little gold content was found in the heart and brain tissues. Similar to the LDGI‐treated group, MSCs‐LDGI group showed high accumulation of gold in liver (7.41 µg g^−1^ of tissue). Compared with LDGI group, a 2.57‐fold decrease of the gold content in the spleen tissue was observed. Interestingly, a 2.34‐fold increase of the gold content was detected in the lungs in the MSCs‐LDGI group. And, the gold content of MSCs‐LDGI in kidney (0.44 µg g^−1^) was much lower than that of the LDGI group. Importantly, the gold content of tumor tissue in the MSCs‐LDGI group was 2.11 µg g^−1^ of tissue, which was 3.35‐fold higher compared to the LDGI group. It showed that MSC–LDGI could be accumulated in tumor tissues more efficiently than LDGI alone. And, both LDGI and MSCs‐LDGI groups had relatively high content of gold in liver. LDGI showed higher accumulate in spleen and kidney. Besides, MSCs‐LDGI had the tendency to accumulate in the lung compared with LDGI group. The ICP‐MS results were consistent with the PA images. However, the nanoparticle‐loaded cells were inevitably accumulated in lung, as shown in the ICP‐MS results (Figure 6b).

The antitumor efficiencies of LDGI, MSCs‐LGI (without DOX), and MSCs‐LDGI (with DOX) via intravenous injection were studied. The tumor bearing mice were intravenously injected with 5 × 10^5^ nanoparticle (LGI or LDGI)‐loading MSCs or 500 µg LDGI when the tumor length reached to 6 mm. The mice were treated with NIR laser (1.5 W cm^−2^, 10 min) post 3 days of administration. The tumor surface temperature was monitored and recorded (Figure 6c). The temperature rising curves showed that after 10 min laser irradiation, the tumor area in the LDGI treated group could reach to 46.3 ± 2.3 °C. In contrast, both of MSCs‐LGI and MSCs‐LDGI groups showed higher temperature rising ability and could reach to 55 ± 3.6 °C post irradiation (Figure 6d), indicating the good photothermal capability of nanoparticles and enhanced accumulation in the tumor by MSCs. To assess the antitumor efficacy, the mice body weight and tumor size were continuously measured in the next 15 days. The body weights of mice showed no significant difference in all the experimental groups (Figure 6e). The tumor volume of mice treated with PBS, DOX, and LDGI, MSCs‐LGI, MSCs‐LDGI without laser irradiation kept increasing after being administrated. The groups of LDGI with laser irradiation had a poor therapeutic efficiency due to the less nanoparticle accumulation via EPR effect. The groups of MSCs‐LGI and MSCs‐LDGI with laser irradiation showed significant anticancer effects. Among the treated groups, MSCs‐LDGI was the most efficient approach to inhibit the tumor growth and recurrence which could be attributed to the enhanced accumulation and combination effect of chemo‐photothermal therapy (Figure 6f). After 15 days post laser irradiation, the picture of tumor for each mouse after executed was recorded (Figure 6g and Figure S14 (Supporting Information)) and the therapeutic efficiency was more visually presented. The results revealed that the MSCs‐LDGI could lead to more efficient treatment and achieve better prognosis. It should be noted that MSCs‐LDGI had the nonspecific accumulation in organs such as lung and liver via intravenous administration. Although intravenous injection had been proven to be an effective approach for MSCs‐LDGI, the administration parameters need to be fully considered and optimized before clinical applications.

## 3 Conclusions

In summary, we designed a light‐responsive plasmonic–magnetic hybrid nanostructure–loaded MSC as a synergistic therapy agent for TNBC. The LDGI‐loaded MSCs with high CXCR4 expression maintained good bioactivity to migrate to the cancer cells in vitro and in vivo. The light‐controlled disassembly of hybrid nanostructures and the corresponding drug release were demonstrated in the stem cells. Furthermore, we found that the secretion of microvesicles containing LDGI from MSCs could be stimulated via laser irradiation, which could facilitate the transportation of LDGI into cancer cells. And, synergistic chemo‐photothermal effect was demonstrated and further explained by the downregulation of Hsp70 expression via the combination treatment. Both in vitro and in vivo experiments showed that the multifunctional therapeutic platform could efficiently inhibit the cancer cell growth. A 4.2‐fold wider intratumor distribution of LDGI was observed in the LDGI‐loaded MSCs compared with the LDGI via local administration. Furthermore, the MSCs‐LDGI's tumor homing ability had been verified by intravenous injection. In contrast to LDGI, a 3.33‐fold enhanced accumulation of nanoparticles in the tumor tissue was observed in the MSCs‐LDGI group via systemic delivery. The MSCs‐LDGI group showed the best therapeutic efficacy via both local and intravenous injection approaches. Future experiments will focus on the magnetic field–guided tumor targeting incorporating with magnetic resonance imaging to realize the multimode imaging and therapy for TNBC. Taken together, the MSCs‐LDGI system offers an effective strategy to overcome the therapeutic resistance and cancer recurrence of breast cancer including TNBC.

## 4 Experimental Section

*Materials and Characterization*: Cetyltrimethylammonium bromide (CTAB), NaBH_4_, ascorbic acid (AA) were purchased from Sigma (USA). HAuCl_4_ • 4H_2_O was purchased from CIVI‐CHEM (China). AgNO_3_ was purchased from Alfa Aesar (UK). HCl, dichloromethane, and diethyl ether were purchased from SCR (Shanghai). The SH—PEG—COOH was purchased from Ponsure Biotechnology (Shanghai). The *N*1‐((ethylimino)methylene)‐*N*3, *N*3‐dimethylpropane‐1,3‐diamine (EDC) and *N*‐hydroxysuccinimide (NHS) were purchased from Aladdin (USA). The DSPC was purchased from TCI (Japan). The mPEG_2000_‐DSPE and DOTAP were purchased from CORDEN PHARMA (Germany). DOX was purchased from MESO (China).

*Preparation of GNR–IO*: The one‐pot seedless method was utilized to prepare CTAB‐coated GNR. Briefly, 50 mL HAuCl_4_ solution (1.0 × 10^−3^ m in distilled water) was mixed with 50 mL CTAB solution (0.2 m in distilled water). Then, 3 mL AgNO_3_ (4.0 × 10^−3^ m) was added to the mixed solution under vigorous stirring. Afterward, HCl (100 µL, 37%) was introduced into the solution to obtain a pH of 1. After this, 0.85 mL AA (85.8 × 10^−3^ m) was added and the flask was stirred intensely until the solution became colorless, then immediately 70 µL freshly prepared NaBH_4_ (0.01 m) was added using ice water. The mixture was shaken for 21 s, and kept in water bath at 30 °C overnight. The color of the solution turned into dark red gradually. The synthesized CTAB–GNRs were purified by centrifugation for 3 times (15 000 rpm, 30 min). Following this, the surfaces of GNR were modified with 5 mL HS—PEG—COOH solution (molecular weight (Mw) = 5000, 0.2 m) via stable the Au—S bond. The reaction was conducted at room temperature for 24 h, then the synthesized GNR—PEG—COOH was purified by centrifugation (15 000 rpm, 30 min) for 3 times with distilled water. The carboxyl‐functionalized GNR were bonded on PEI‐modified IO using EDC and NHS.

*Loading DOX into LDGI*: The nanoparticle preparation was referred to reverse phase evaporation method. Three kinds of lipids, DSPC, mPEG_2000_‐DSPE, and DOTAP were used in this procedure. First, the lipid mixtures of DOTAP/mPEG_2000_‐DSPE/DSPC (50/45/5 mole ratio) were dissolved in 3 mL dichloromethane and diethyl ether (volume ratio = 1:1) in a round‐bottom flask. Meanwhile, 4 mg GNR–IO and 0.5 mg DOX in water solution were sonicated for 5 min to get a uniform dispersion. Afterward, GI and DOX mixture were introduced into lipids sonicating for 30 min to realize emulsification for the next step. Then, the organic solvent was removed using rotary evaporator at 40 °C until the gel phase disappeared. 1 mL water was added into the system to ensure that organic solvent was wiped out completely. And, the product was purified 3 times with water to get rid of free DOX and lipids. The LDGI was stored at 4 °C for tests.

*Characterization*: The morphology information of nanoparticles was obtained by TEM (JEM‐1230, JEOL Ltd., Japan) with an accelerating voltage of 100 kV. The samples were prepared on the copper grid by dropping a concentrated nanoparticle solution (10 µL) and left for 30 min to dry on the grids in room temperature. The absorption spectra of GNR, IO, and LDGI were determined by UV–vis spectroscopy (Cary 60 UV‐Vis, Agilent Technologies). The fluorescence spectra of 120 µg mL^−1^ LDGI solution and 1 µg mL^−1^ DOX solution in water were taken on a Cary Eclipse Fluorescence Spectrophotometer (Agilent Technologies). And, the surface zeta potential and hydrodynamic size of nanoparticles were evaluated in deionized water, using a Malvern Zetasizer Nano ZS90.

*Temperature Rising Curve*: Continuous‐wave NIR laser (808 nm, ANJ, Beijing) was chosen for PTT. The laser irradiation was performed with different laser powers (1, 1.5, 2, 2.5, 3 W cm^−2^) and different LDGI concentrations (15, 30, 60, 120 µg mL^−1^) for 15 min. The laser spot was adjusted to cover the entire bottle. The temperature increases and thermal images were recorded with infrared thermal camera (DALI, China) at each minute. The temperature rising ability of MSCs‐LDGI was recorded by infrared thermography each minute for 20 min. Then, the data were sorted and plotted as time–temperature curve.

*Cell Line and Culture*: The mesenchymal stem cells used in the research were isolated from human umbilical cord, and were cultured in regular growth medium consisting of low‐glucose Dulbecco's modified Eagle's medium (DMEM) (Gibco) supplement with 10% fetal bovine serum (FBS) (Gibco), penicillin (100 U mL^−1^), streptomycin (100 µg mL^−1^), and growth factor GbFb (10 ng mL^−1^). All the experiments were carried out with human mesenchymal stem cells (hMSCs) of the fifth to tenth passage. Human triple negative breast cancer cell line MDA‐MB‐231 cells were cultured in high‐glucose DMEM (Gibco) supplement with 10% FBS (Hyclone, Thermo Scientific, USA), penicillin (100 U mL^−1^), and streptomycin (100 µg mL^−1^). Then, cells were incubated at 37 °C in a humidified atmosphere containing 95% air and 5% CO_2_.

*LDGI‐Loading Studies*: MSCs were incubated with DMEM medium containing 120 µg mL^−1^ LDGI for 24 h in the incubator at a density of 50 000 cells mL^−1^. And, rinsed with PBS (Hyclone) for 3 times to remove the nanoparticles that were not uptaken by MSCs, and then, the fresh medium for further experiment was added. Prussian Blue Kit (Solarbio, Beijing) was used to stain the IO. MSCs were incubated with LDGI in different concentrations (15, 30, 60, 120 µg mL^−1^) for 24 h. After rinsing with PBS for 3 times, MSCs‐LDGI were fixed with 4% paraformaldehyde for 20 min, then cells were immersed with Perls stain solution for 30 min, after rinsing with water for 5 min, cells were immersed with fast red for another 10 min.

*ICP‐MS of MSCs‐LDGI*: MSCs were conducted in 24‐well plate with a density of 1 × 10^4^ cells per well. In this analysis, the control group was MSC only. To quantify the amount of gold and iron atoms in MSCs‐LDGI, the cells were rinsed with PBS for 3 times and collected at the time points of 4, 12, 24 h. The cell plates were lysed with aqua regia solution (concentrated hydrochloric acid:concentrated nitric acid = 3:1 v:v) to digest LDGI. The results were quantified by ICP‐MS (Thermo Fisher, iCAP Q).

*In Vitro Migrating Assay*: Cell migration ability was studied in vitro by wound healing experiment. MSCs‐LDGI were prepared in serum‐free medium as mentioned above, and 70 µL MSCs‐LDGI (50 000 cells mL^−1^) was seeded in one of the 2‐well culture insert (ibidi, Germany), cancer cells under the same concentration were seeded in another well. MSCs were used as control. After the cells were cultured for 24 h, the insert was gently removed and the gap distance between stem cells and cancer cells was recorded.

*CXCR4 Expression on MSCs*: MSCs were incubated with nanoparticles (GNR, IO, and LDGI) for 2 h, after rinsed with PBS for 3 times to remove the free nanoparticles. MSCs loaded with nanoparticles were cultured with fresh medium for another 22 h. Following this, MSCs were collected and the total protein of stem cells was collected by lysing the cells with a Cell lysis buffer for Western and IP kit (Beyotime). The expression of CXCR4 was examined by western blot.

*Temperature Rising Ability of MSCs‐LDGI*: MSCs were planted in well plate 1 day before incubation with LDGI at different concentrations (15, 30, 60, 120 µg mL^−1^). After being cultured for 24 h, free LDGI was rinsed by PBS for 3 times. Then, the MSCs‐LDGI with different LDGI culture concentrations were irradiated by laser (3 W cm^−2^, 20 min). The temperature values were recorded by infrared thermal camera.

*MSC Cell Viability Test by CCK‐8 Assay*: The cells were planted in 96‐well plate overnight before incubation with LDGI, the concentration of LDGI and LGI was 15, 30, 60, 120 µg mL^−1^. And, the DOX concentrations were calculated corresponding to LDGI of respective groups. After 24 h incubation, MSCs‐LDGI were rinsed with PBS for 3 times, and the laser group was irradiated with laser (3 W cm^−2^, 5 min). After incubating for another 4 h, the cell viability of MSCs was tested by CCK‐8 assay.

*Calcein‐AM/PI Staining Assay*: After being treated, the cells were stained by calcein‐AM solution for 30 min, and rinsed with PBS for 3 times. Then, the cells were stained by PI solution for 10 min. After rinsing with PBS for 3 times, the cells were observed via EVOS system (Life Technologies, USA).

*DOX Release Triggered by Photothermal Effect*: The behavior of DOX release from LDGI with irradiation was investigated. This experiment was evaluated in deionized water with LDGI concentration of 120 µg mL^−1^. The LDGI solution was irradiated with laser power of 3 W cm^−2^ for 3 min. The DOX‐released behavior was investigated by fluorescence spectroscopy.

*Laser Scanning Confocal Microscopy*: The laser triggered release of DOX in cellular level was further observed using laser scanning confocal microscope (Leica TCS SC5 II). MSCs were planted into confocal dish with density of 10^5^ cells per dish 24 h before adding LDGI. MSCs were treated with LDGI (120 µg mL^−1^) for 4 h, and rinsed with PBS. The laser group was irradiated for 5 min (3W cm^−2^), and incubated for another 2 h. Then, the cells were fixed with 4% paraformaldehyde, and the cell lysosomes were stained with Lysol‐Tracker Green for 20 min, after rinsing with PBS, the nucleus was stained with DAPI for 10 min. Then, the cells were used for confocal. The confocal of MBA‐MD‐231 cells and MSCs was done in the same method. After planting the MSCs‐LDGI and cancer cells with a ratio of 2:1, the cells were cocultured for 24 h before staining.

*In Vitro Antitumor Effect of LDGI*: The MSCs were incubated with 120 µg mL^−1^ LDGI for 24 h and MSCs‐LDGI were cocultured with MDA‐MB‐231 cells into 96‐well plate with different cells ratios of 4:1, 2:1, 1:1, 1:2, and 1:4. After 24 h incubation, the cells were irradiated with laser (3 W cm^−2^, 5 min), and the cell viabilities were examined by CCK‐8 assay and calcein‐AM/PI staining assay.

*Tumor Model of Mice*: Female BALB/c athymic nude mice aging 5 weeks were purchased from SLAC, the mice were raised in specific pathogen free animal house at the Tongji University. Animal care and all of the mice experiments obeyed the rules of the Institutional Animal Care and Use Committee of the Tongji University. The tumor model was established using the negative breast cancer cell line MDA‐MB‐231. The MDA‐MB‐231 cells were harvested and suspended in 100 µL PBS Matrigel mixture containing 1 × 10^6^ MDA‐MB‐231 cells and injected into the subcutaneous dorsa of mice. The tumor size and mice weight were measured by digital calipers and weight scale. Treatment was set out when tumor reached about 6 mm in length and width.

*Intratumor Distribution (Local Injection)*: MSCs were cultured with 120 µg mL^−1^ LDGI for 24 h. After rinsing cells with PBS for 3 times, MSCs‐LDGI were collected and injected intratumorally into nude mice (10^5^ cells per mouse). After 3 days, photoacoustic imaging was used to value the MSCs‐LDGI distribution in the tumor tissue.

*Intravenous Administration*: MSC‐treated procedures were same with local injection. After collecting, the MSCs‐LDGI were slowly intravenously injected into the tail vein of mice for 3 min (5 × 10^5^ cells, 200 µL). The investigations of distribution and treatment were done after 3 days administration.

*PA Imaging Analysis*: After being administrated with nanoparticles for 3 days, the nanoparticle biodistributions were monitored by PA imaging. The equipment was independently set by Prof. Qian Cheng' lab (Tongji University). The wavelength of pulsed laser being used in the experiment was 820 nm, and the power density was 20 mJ cm^−2^.

*Antitumor Efficacy Experiments*: The mice were randomly divided into nine groups of three to five mice each group, minimizing the differences of weights and tumor sizes in each group. The mice were intratumorally administered with a) PBS, b) PBS and laser treatment, c) DOX (1 µg of DOX), d) LDGI (100 µg of LDGI per mouse), e) LDGI and laser treatment (100 µg LDGI per mouse), f) MSCs‐LGI (10^5^ MSCs per mouse), g) MSCs‐LGI and laser treatment (10^5^ MSCs per mouse), h) MSCs‐LDGI (10^5^ MSCs, 100 µg per mouse), i) MSCs‐LDGI and laser treatment (10^4^ MSCs). The mice were intravenously administered with a) PBS, b) DOX (5 µg of DOX), c) LDGI (500 µg of LDGI per mouse), d) LDGI and laser treatment (500 µg LDGI per mouse), e) MSCs‐LGI (5 × 10^5^ MSCs per mouse), f) MSCs‐LGI and laser treatment (5 × 10^5^ MSCs per mouse), g) MSCs‐LDGI (5 × 10^5^ MSCs, 500 µg per mouse), h) MSCs‐LDGI and laser treatment (5 × 10^5^ MSCs). After 3 days, the tumors were irradiated with NIR light (1.5 W cm^−2^, 10 min). The temperature was recorded using an infrared camera thermographic system. The tumor size and mice weight were measured by digital calipers and weight scale, and the tumor volumes were calculated based on the following formula: $V\;=\;\frac{\pi \;\times \;LW^{2}}{6}\;$. In this equation, *V*, *L*, and *W* are the volume, length, and width of tumor, respectively. The relative tumor volume was calculated as *V*/*V*_0_, where *V*_0_ is the tumor volume at initiation of the treatment. At 15th day, all the animals were euthanized, and the tumors were dissected and weighed.

*Statistical Analysis*: The result was given as mean ± standard error of mean (SEM). To describe statistical differences, the test was used. Differences were considered statistically significant for *p* < 0.05 (*p* < 0.05 (*), *p* < 0.01 (**), *p* < 0.001 (***)).

## Conflict of Interest

The authors declare no conflict of interest.

## Supporting information

Supplementary
