# Supplementary material for: A Light‐Triggered Mesenchymal Stem Cell Delivery System for Photoacoustic Imaging and Chemo‐Photothermal Therapy of Triple Negative Breast Cancer
Source: Adv Sci (Weinh). 2018 Aug 24;5(10):1800382. doi: 10.1002/advs.201800382 (PMC6193170; doi:10.1002/advs.201800382)
Supplement: Supplementary file 1 — Supplementary [file ADVS-5-1800382-s001.pdf]

Copyright WILEY-VCH Verlag GmbH & Co. KGaA, 69469 Weinheim, Germany, 2018.

ADVANCED SCIENCE
Open Access

Supporting Information

for *Adv. Sci.,* DOI: 10.1002/advs.201800382

A Light-Triggered Mesenchymal Stem Cell Delivery System for Photoacoustic Imaging and Chemo-Photothermal Therapy of Triple Negative Breast Cancer

Chang Xu, Qishuai Feng, Haocheng Yang, Guangxue Wang, Liqun Huang, Qianwen Bai, Chuyi Zhang, Yilong Wang, Yingna Chen, Qian Cheng, Mengwei Chen, Yu Han, Zuoren Yu,* Maciej S. Lesniak, and Yu Cheng**

Supporting Information

A Light-Triggered Mesenchymal Stem Cell Delivery System for Photoacoustic Imaging and Chemo-Photothermal Therapy of Triple Negative Breast Cancer

Chang Xu[a], Qishuai Feng[a], Haocheng Yang[a], Guangxue Wang[b], Liqun Huang[c], Qianwen Bai[a], Chuyi Zhang[b], Yilong Wang[a], Yingna Chen[d], Qian Cheng[d], Mengwei Chen [a], Yu Han[e], Zuoren Yu[b]*, Maciej S. Lesniak[e], Yu Cheng[a]**

[a] The Institute for Translational Nanomedicine, Shanghai East Hospital; The Institute for Biomedical Engineering & Nano Science, Tongji University School of Medicine, 150 Jimo Road, Shanghai, 200120, China.

[b] Research Center for Translational Medicine, Key Laboratory of Arrhythmias of the Ministry of Education of China, East Hospital, Tongji University School of Medicine, 150 Jimo Road, Shanghai 200120, China.

[c] Department of Urology, Shanghai East Hospital, Tongji University School of Medicine, Shanghai, 200120, China.

[d] Institute of Acoustics, Tongji University, Siping Road 1239, Shanghai 200092, China.

[e] Department of Neurological Surgery, The Feinberg School of Medicine, Northwestern University, Chicago, IL 60611, USA.

Corresponding authors: yilongwang@tongji.edu.cn; zuoren.yu@tongji.edu.cn; yucheng@tongji.edu.cn

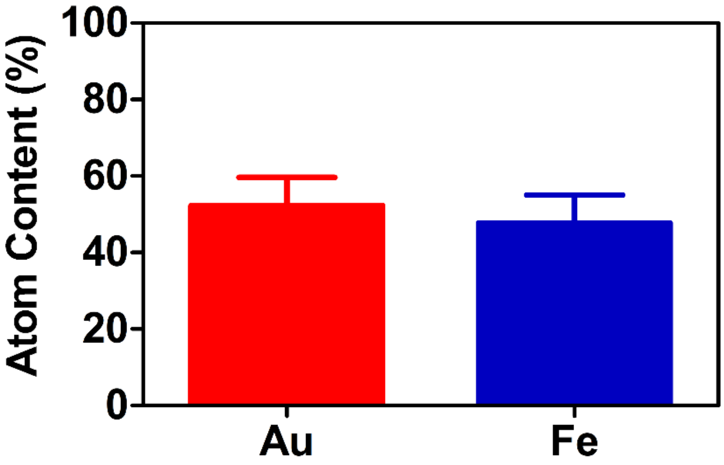

Figure S1. Au and Fe atom contents in LDGI.

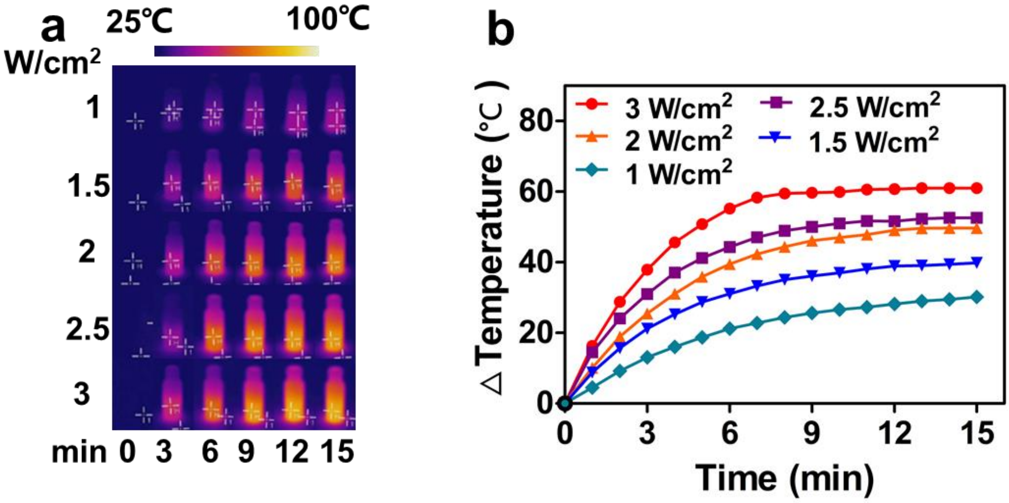

Figure S2. Photothermal analysis with different laser power. (**a**) Photothermal effect of 120 μg/mL LDGI with different laser irradiation power (1 W/cm^2, 1.5 W/cm^2, 2 W/cm^2, 2.5 W/cm^2, 3 W/cm^2) and corresponding temperature changing curves (**b**) under 808 nm laser at 3 W/cm^2 for 15 min.

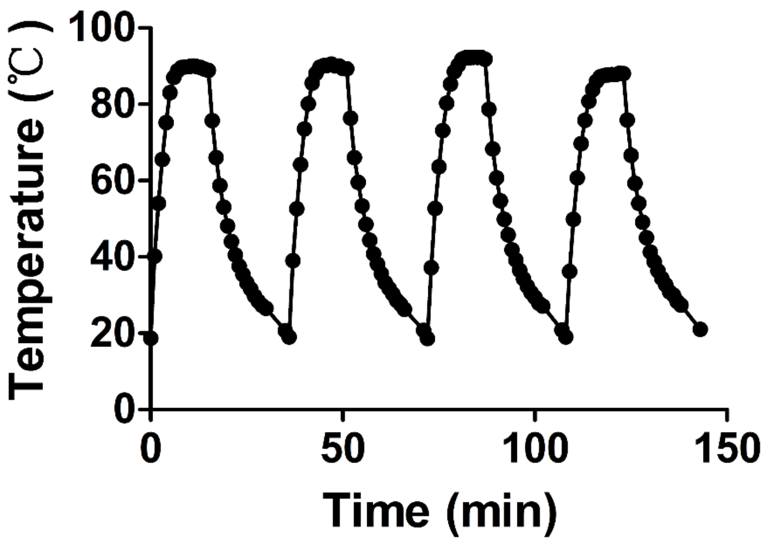

Figure S3. Photothermal ability of LDGI for four circles.

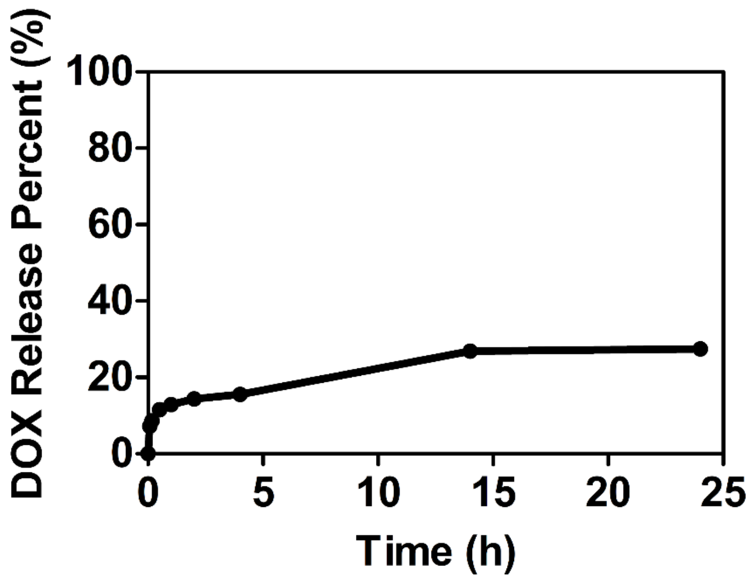

Figure S4. Cumulative DOX release curve within 24 h after synthesis of LDGI.

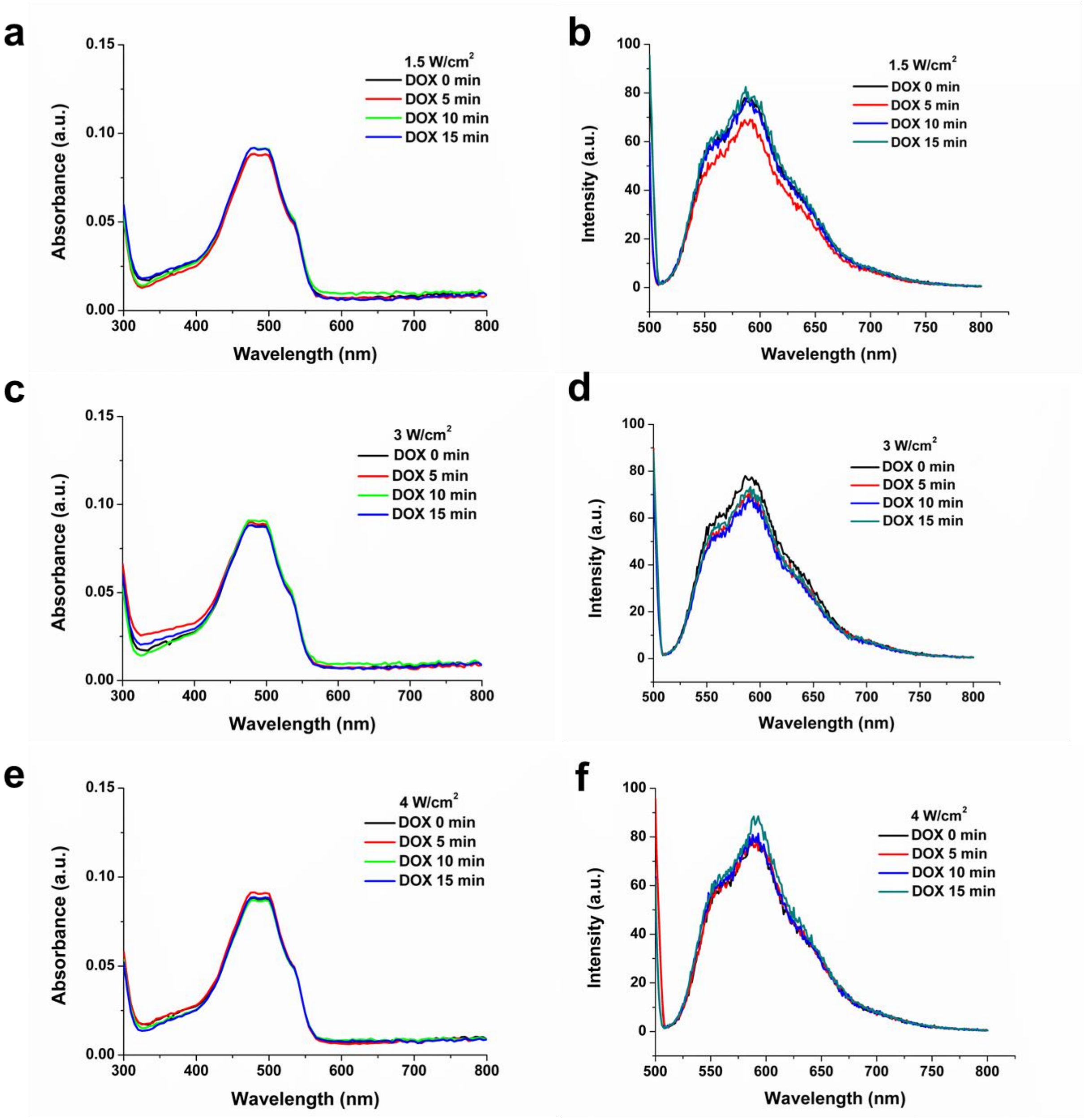

Figure S5. Time-dependent (5 min, 10 min and 15 min) influence of DOX under 808 nm laser irradiation in different powers. (**a**) DOX absorbance in UV-vis-NIR and (**b**) fluorescence spectra with laser irradiation of 1.5 W/cm^2, (**c**) DOX absorbance in UV-vis-NIR and (**d**) fluorescence spectra with laser irradiation of 3 W/cm^2 and (**e**) DOX absorbance in UV-vis-NIR and (**f**) fluorescence spectra with laser irradiation of 4 W/cm^2. (The initial concentrations of DOX was 10 μM.)

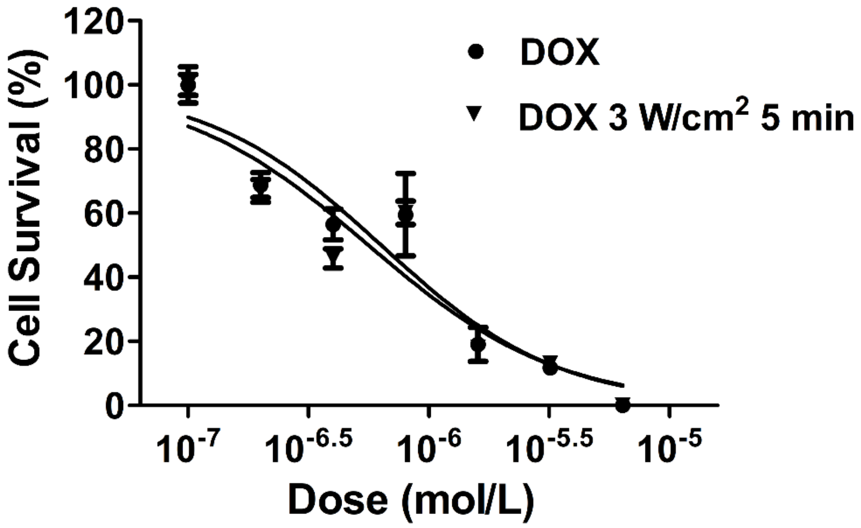

Figure S6. IC50 curve of MDA-MB-231 cells treated with DOX exposed to laser (3 W/cm2, 5 min).

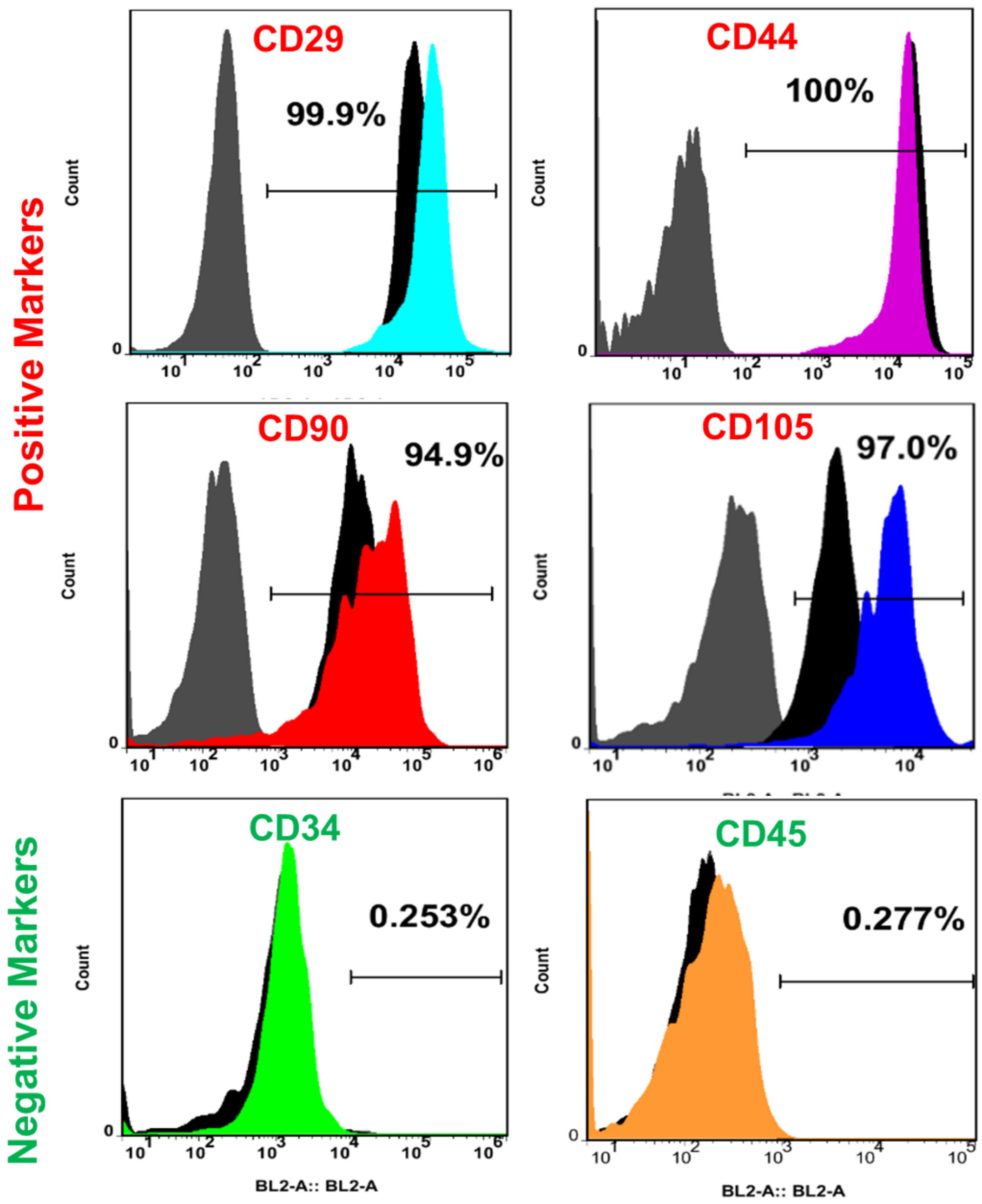

Figure S7. Specific surface markers of stem cells expression on MSCs treated with LDGI after 72 h tested by Flow cytometry.

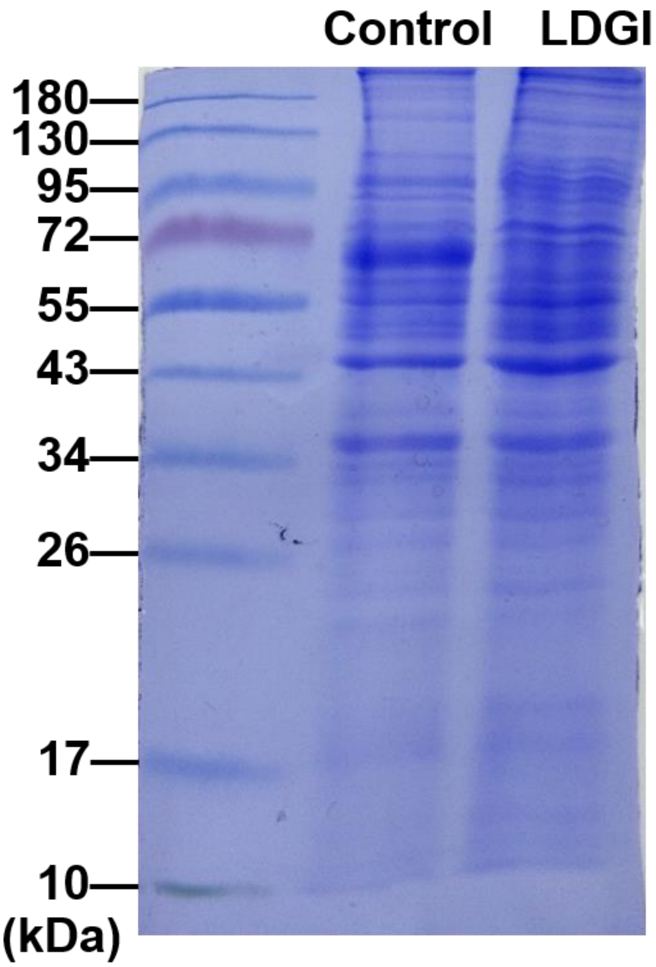

Figure S8. SDS-PAGE protein analysis. Samples were stained with Coomassie brilliant blue kit. MSCs-LDGI supernatant extracellular vesicles were collected 24 h after incubation, and MSCs supernatant extracellular vesicles were collected as control.

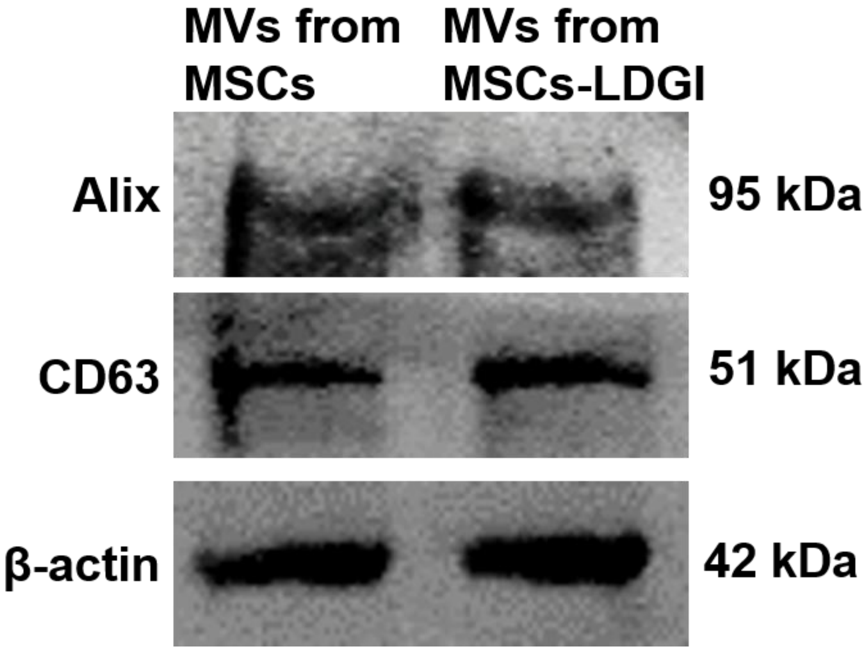

Figure S9. Cell exsosome protein expression on MVs from MSCs and MSCs-LDGI, respectively.

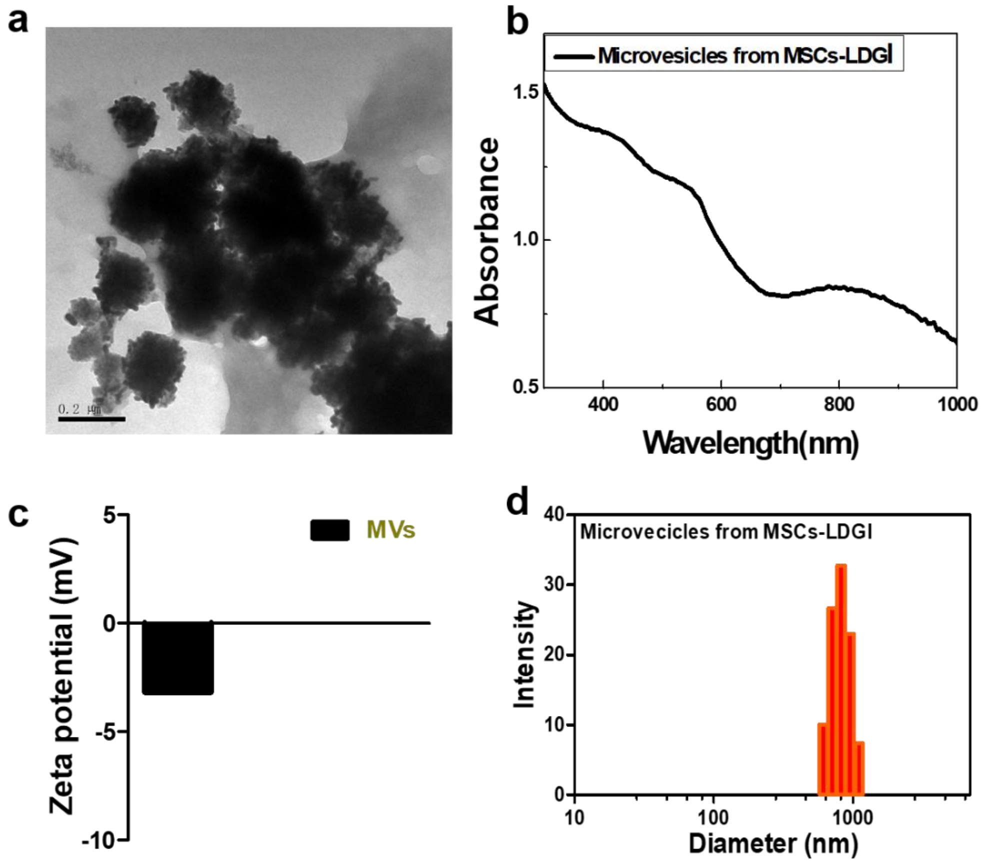

Figure S10. Characterization of microvesicles from MSCs-LDGI. (a) Representative TEM image of microvesicles from MSCs-LDGI. (b) UV–vis-NIR spectra of microvesicles from MSCs-LDGI. (c) Zeta potential of microvesicles from MSCs-LDGI in PBS. (e) Size distribution of microvesicles from MSCs-LDGI in PBS.

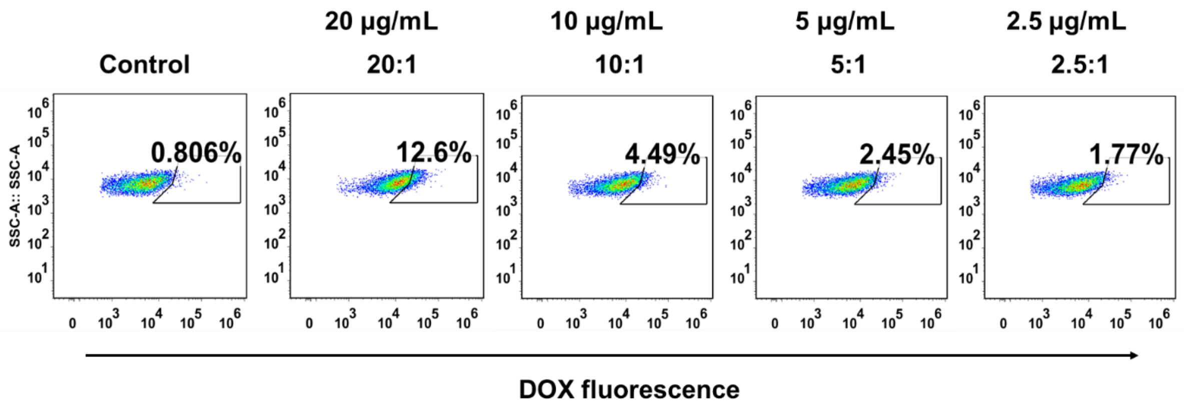

Figure S11. DOX fluorescence of MDA-MB-231 cells treated with MVs from MSCs-LDGI with different concentrations (20:1, 10:1, 5:1, 2.5:1).

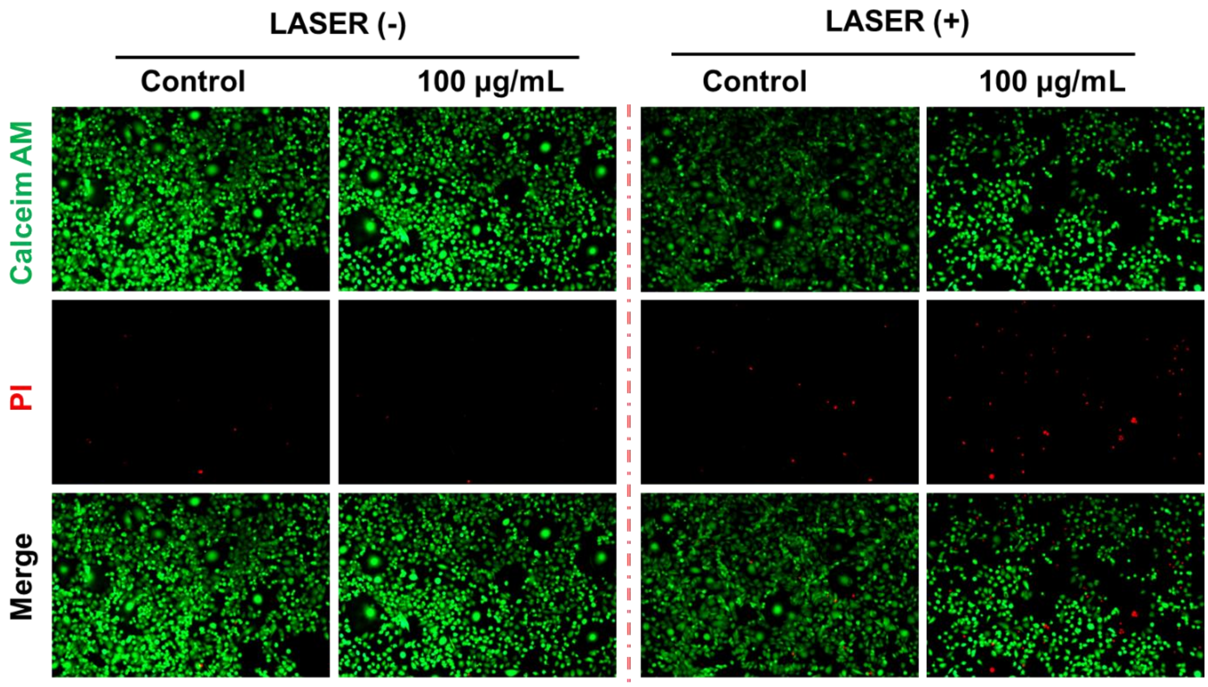

Figure S12. LIVE/DEAD cell viability tests of MDA-MB-231 cells treated with MVs from MSCs-LDGI with and without laser irradiation (3 W/cm^2).

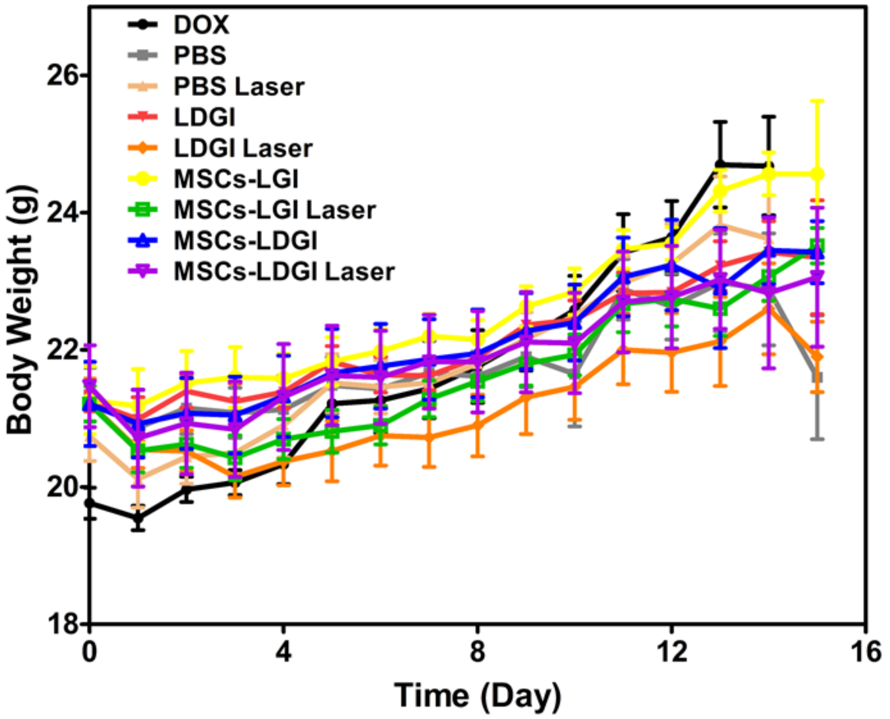

Figure S13. Body weight curves of mice intratumorally injected with DOX, PBS, LDGI, MSCs-LDI, MSCs-LDGI with and without NIR laser.

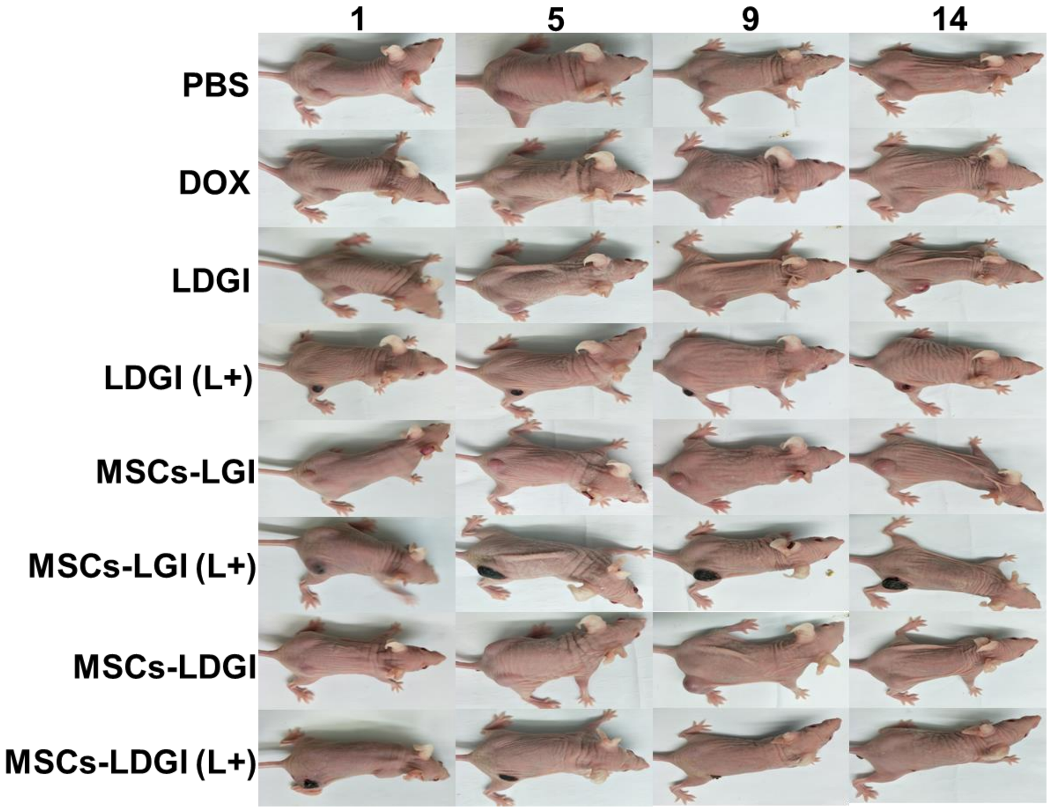

Figure S14. Images of mice of intravenous injected with DOX, PBS, LDGI, MSCs-LDI, MSCs-LDGI with and without NIR laser irradiation within 15 days.
